# Incorporation of Nanoparticles in Coatings on Acrylic Resin: Impact on Wettability and Antifungal Action

**DOI:** 10.3390/ma19102130

**Published:** 2026-05-19

**Authors:** Juliana de Freitas Gouveia Silva, Lady Daiane Pereira Leite, Tiago Moreira Bastos Campos, Cristiane Yumi Koga-Ito, Gilmar Patrocínio Thim, Tarcisio José de Arruda Paes Junior

**Affiliations:** 1 Dental Materials Laboratory, Department of Dental Materials, Institute of Science and Technology, São Paulo State University (UNESP), São José dos Campos, São Paulo 12245-000, Brazil; 2 Genome Laboratory, Department of Environmental Engineering, Institute of Science and Technology, São Paulo State University (UNESP), São José dos Campos, São Paulo 12220-290, Brazil; 3 Plasma and Processes Laboratory (LPP), Aeronautics Institute of Technology (ITA), São José dos Campos, São Paulo 12228-900, Brazil

**Keywords:** wettability, acrylic resin, nanoparticles, titanium dioxide, zinc oxide

## Abstract

**Highlights:**

**What are the main findings?**

- ZnO and TiO_2_ nanoparticles reduced *Candida albicans* hyphal formation on acrylic resin.
- Nanoparticles did not affect fungal proliferation on heat-cured acrylic resin.
- ZnO and TiO_2_ improved surface wettability without altering mechanical properties.

**What are the implications of the main findings?**

- Nanoparticle-modified resins may limit fungal pathogenicity on denture surfaces.
- Improved wettability may enhance complete denture retention clinically.
- Mechanical property of acrylic resin is preserved after nanoparticle addition.

**Abstract:**

Acrylic resin is widely used in the fabrication of complete dentures, interacting significantly with the intraoral environment. However, complete dentures face challenges such as stability issues and biofilm accumulation. Glaze application is a common method to reduce surface porosity and microbial adhesion, but it also decreases surface wettability, potentially impairing salivary film formation essential for peripheral sealing. This study aimed to incorporate titanium dioxide and zinc oxide nanoparticles into the glaze applied to thermally activated acrylic resin (TAAR) via spray coating to enhance surface wettability and antifungal activity. Four groups were tested: G (TAAR + commercial glaze − control); AlG (TAAR + commercial glaze + aluminum oxide − roughness control); TiG (TAAR + commercial glaze + titanium dioxide); and ZnG (TAAR + commercial glaze + zinc oxide). Evaluations included flexural strength, color and translucency, surface analysis and antibiofilm activity against *Candida albicans*. Data were analyzed using one-way ANOVA. No statistically significant differences in mechanical strength (MPa) were observed (G: 108.54 ± 8.36; AlG: 113.60 ± 11.95; ZnG: 111.98 ± 9.27; TiG: 113.66 ± 10.41). Surface roughness significantly increased, and contact angle decreased, indicating improved wettability. Regardless of the antifungal activity no improvement was detected (G: 6.71 ± 0.10; AlG: 6.82 ± 0.08; ZnG: 6.72 ± 0.20; TiG: 6.66 ± 0.18). In conclusion, the incorporation of nanoparticles into the glaze improves the wettability of acrylic resin surfaces, potentially enhancing peripheral sealing and denture retention, which is beneficial for patients with reduced alveolar ridge height.

## 1. Introduction

Acrylic resin interacts with the oral environment in ways that favor biofilm accumulation, as its polymeric nature and high surface energy promote the adhesion of organic matter and subsequent microbial growth. Despite these drawbacks, it remains the material of choice for total prostheses due to its biocompatibility and adaptability in completely edentulous patients. However, biofilm formation on its surface and insufficient surface wettability can compromise prosthesis stability in the oral cavity [1,2].

The stability of a total prosthesis partly depends on the formation of a thin salivary film between the internal surface and soft tissues, which creates a peripheral seal. This mechanism is influenced by surface irregularities and surface energy. The incorporation of nanoparticles, by modifying surface roughness, can enhance peripheral sealing and improve retention, particularly in prostheses with narrow or low borders [3]. In a previous study, nanoparticles were embedded in a glaze (liquid medium); however, no antimicrobial activity or improvement in wettability was observed, as the nanoparticles became coated by the liquid, which prevented direct contact with fungi and limited the increase in surface area [4].

Different types of nanoparticles (NPs) have shown potential to increase superficial wettability and, in some cases, provide antimicrobial effects. Zinc oxide nanoparticles (ZnO), for example, are active against both Gram-positive and Gram-negative bacteria as well as the fungus *Candida albicans*. Their antimicrobial performance depends on size, morphology, and concentration, with smaller particles and higher concentrations generally showing greater efficacy due to enhanced membrane penetration and intracellular access [1,5,6,7].

Titanium dioxide (TiO_2_) has also attracted attention for its antimicrobial properties against a wide spectrum of bacteria and fungi. Its mechanism involves generating reactive oxygen species (ROS), which damage cell walls and disrupt cytoplasmic membranes. Structurally, TiO_2_ nanoparticles adopt an octahedral form, where six oxygen anions are shared among three titanium cations (IV), a feature that contributes to their antimicrobial efficiency [7,8,9,10,11,12,13].

Current research emphasizes strategies to improve the superficial wettability of acrylic resin in total prostheses, thereby increasing stability. At the same time, incorporating nanoparticles with antifungal activity, particularly against *C. albicans*, offers a promising approach to preventing prosthetic candidiasis and enhancing oral health (Table 1).

The objective of this study was to enhance the surface wettability and antifungal properties of thermally activated acrylic resin used in the fabrication of complete dentures. By incorporating titanium dioxide and zinc oxide nanoparticles into the glaze coating, applied via spray coating, the research aims to address the challenges posed by biofilm accumulation and stability issues faced by complete dentures. The four experimental groups studied are aimed at evaluating the impact of different nanoparticle additions on surface characteristics. Through a comprehensive analysis involving material characterization tests, flexural strength assessments, color and translucency measurements, and antibiofilm analysis against *C. albicans*, the study seeks to determine the effectiveness of nanoparticle incorporation in improving surface properties, retention of complete dentures, without compromising biofilm proliferation.

## 2. Materials and Methods

### 2.1. Experimental Design

**Figure 1:**
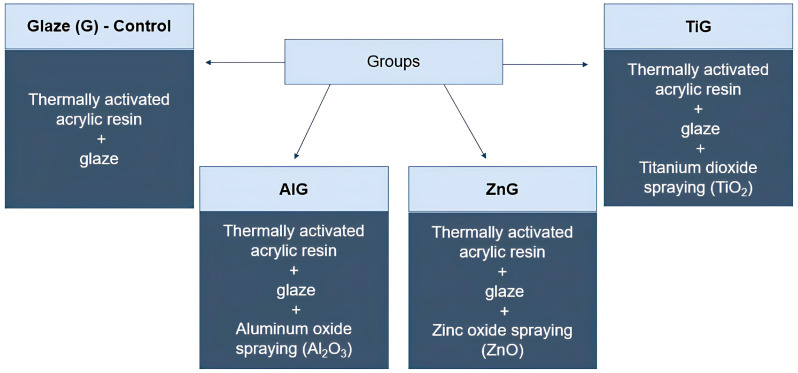
Experimental groups.

**Figure 2:**
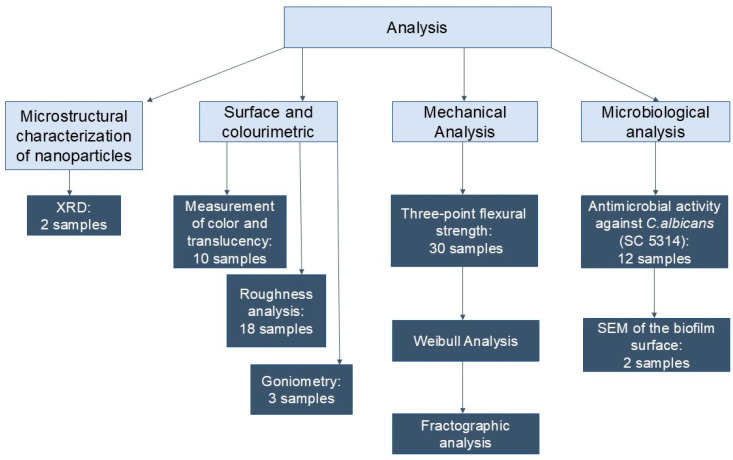
Analysis performed.

Experimental groups and analyses performed are described in Figure 1 and Figure 2.

### 2.2. TiO_2_, ZnO and Al_3_O_2_ Nanoparticles

The TiO_2_ and ZnO nanoparticles used were obtained from Sigma-Aldrich (St. Louis, MO, USA), with a size of 50 nm. The Al_3_O_2_ nanoparticles used were from Alcoa (Pittsburgh, PA, USA) and had a size of 100 nm. Al_3_O_2_ nanoparticles were used as a control for roughness.

#### Microstructural Characterization of Nanoparticles

The crystalline phases of the TiO_2_, ZnO, and Al_3_O_2_ nanoparticles were characterized by X-ray diffraction (XRD), which was conducted to visualize the crystalline phase of the nanoparticles through molecular diffraction. It was performed using a Philips X’Pert da PANalytical equipped with Cukα radiation, operating at 45 kV and 40 mA, in the range of 10° ≤ 2θ ≤ 90°, Δθ = 0.02°, and a time of 30 s per Δθ. This analysis was performed at the Materials Characterization Laboratory (LabMat) of the Aeronautics Institute of Technology (ITA).

### 2.3. Sample Preparation for TAAR

For the application of nanocomposites on the surface of thermally activated acrylic resin (TAAR), 76 disk samples of 10 × 2 mm and 30 samples in the form of bars (30 × 10 × 3 mm) of acrylic resin were fabricated. Both the disk and bar standards were fabricated and included in a plastic flask, gypsum, and a wall of a laboratory silicone-based molding material (Rodorsil—VWL, Elkem, Oslo, Norway).

Type II gypsum was spatulated in the following proportions. The metal standards were equidistant and parallel to one another. After the final gypsum set, a wall of laboratory dense mass condensation silicone was fabricated around the standards. A thin layer of insulating material, Al-Cote (Dentsply Ind. Com. Ltda, New York, NY, USA) for gypsum, and acrylic resin was applied, and gypsum was added again to completely fill the flask. After one hour of the final inclusion phase, the flask was opened, and the metal standards were completely removed.

For the pressing procedure, two portions of thermally activated clear acrylic resin (VipiCril Dental, Dentsply Ind, New York, NY, USA) in the transition from its fibrillar to plastic phase were manipulated, and the resin mass was accommodated in the flask in the spaces left by the standards. The flask was pressed at 1.0 Kgf until the acrylic resin flowed through the edges of the flask, maintaining the appropriate pressure while screwing the parts of the flask.

The powder/liquid ratio of the resin was 15 g and 7.5 mL. Regarding the polymerization cycle, the flask was positioned in an 800 W microwave (Continental AW-30, Curitiba, Brazil); then, a cycle of 20 min with 20% power was performed, followed by an additional 5 min with 60% power.

The flask was kept at room temperature for approximately two hours until complete cooling was achieved. After opening the flasks, the specimens were initially stored in distilled water during the finishing session. To finish the pieces, a polisher (Ecomet 250, Buehler, São José dos Campos, Brazil) was used under constant water cooling.

The glaze used was Palaseal (Heraeus Kulzer-Jelenko) with methyl methacrylate because it has a low viscosity, facilitating its application on the sample surface. The glaze was applied with or without the incorporation of nanoparticles and then photopolymerized. For the control group, glaze was applied as determined by the manufacturer, using a microbrush in two subsequent layers. The sample was photopolymerized using a photopolymerizer with 1000 mW/cm^2^ power for 20 s on each side (VALO Grand, Ultradent Products, South Jordan, UT, USA).

### 2.4. Incorporation of Nanocomposites into Glaze

TiO_2_, ZnO, and Al_3_O_2_ nanoparticles were incorporated into the Palaseal glaze (Heraeus Kulzer-Jelenko, Hanau, Germany) using the particle spraying technique through a sieve to partially expose them, aiming to facilitate the release and action of antimicrobial activity.

For the nanoparticle addition groups, glaze was applied to the specimen as determined by the manufacturer. Spraying was then performed using a sieve with a particle size of 75 µm, mesh size of 200 TY, 10 mm from the sample surface and perpendicular to it, during the period of 15 s.

**Figure 3:**
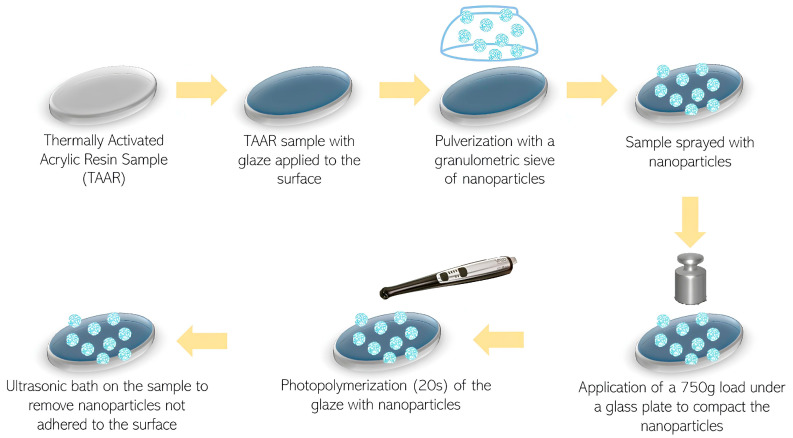
Sample preparation scheme.

The nanoparticles were pressed onto the resin surface using a glass plate at a constant force of 750 g. This application was performed twice to ensure that the entire surface of the sample was covered with nanoparticles. The nanoparticle-glaze assembly was then photopolymerized using a photopolymerizer with 1000 mW/cm^2^ power for 20 s on each side of the sample (VALO Grand, Ultradent Products) (Figure 3).

### 2.5. Color and Translucency Measurement

For color and translucency analyses, specimens with a thickness of 2 mm (*n* = 20), similar to the thickness of the prosthesis, were used. A Konica Minolta colorimeter spectrophotometer (CM 2600, Tokyo, Japan) and a system for measuring the optical properties according to CIE-Lab (International Commission on Illumination, L*, a*, b*) were utilized. The measurements were coordinated using a CIE standard 2°observer and CIE standard illuminant D65.

Before each specimen was measured, the colorimeter spectrophotometer was calibrated according to the manufacturer’s recommendations. Color differences were defined as the differences in color coordinates between the samples, black background, and white background, which are known as Deltas (Δ). The deltas for L* (ΔL), a* (Δa), and b* (Δb) can be negative (−) or positive (+). The color difference was mathematically calculated and is denoted by ΔE*. To determine the total color difference between the three coordinates, equation (Equation (1)) was used:

(1) $$∆E^{*}={\left[{∆L}^{*2}+{∆a}^{*2}+{∆b}^{*2}\right]}^{\frac{1}{2}}$$

The parameters “L*,” “a*” and “b*” correspond, respectively, to lightness, redness and greenness (modelo CIE, Commision Internationale de l’Eclairage), with black backgrounds (L* = 1.8, a* = 1.3, b* = −1.5) and white backgrounds (L* = 95.7, a* = −1.3, b* = 2.6).

The translucency alteration (Tp, translucency parameter) was determined by reading the difference between white and black backgrounds. (Equation (2))

(2) $$T_{P}={\left[{{{\left(L_{p}*-L_{b}*\right)}^{2}+\left(a_{p}*-a_{b}*\right)}^{2}+\left(b_{p}*-b_{b}*\right)}^{2}\right]}^{\frac{1}{2}}$$

The subscripted letters “*p*” refer to color coordinates on the black background and the subscripted letters “*b*” refer to the white background. The higher the value of *Tp*, the higher the translucency.

The color coordinates of the backgrounds were white (L: 84.95; a: −0.38; b: 2.93) and black (L: 25.58; a: −0.15; b: −0.24).

The test was performed with the treated surface of the sample facing away from the light, considering that this face will clinically be in contact with the oral mucosa on the inner part of the complete denture. Thus, color analysis was performed on the external, polished, and smooth sides of the acrylic resin.

### 2.6. Roughness Analysis

A roughness tester (Surftest SJ 400, Mitutoyo, Tokyo, Japan) was used. The surface roughness (*n* = 10) of the working surface of each sample was analyzed using the parameters Ra (average absolute roughness of the heights of the irregularities along the profile; height parameter) and Rz (average roughness depth). The specimens were individually identified, and on the surface of each sample, six parallel readings of 3 mm each were taken equidistantly, with a scanning speed of 0.2 mm/s, using a Gaussian filter and a cut-off value of 0.8 mm.

### 2.7. Goniometry

The contact angle analysis test was performed using a goniometer (Theta Lite model, Attension, Biolin Scientific, Espoo, Finland) under controlled temperature and connected to a computer using One Attention software 1.8. A drop of distilled water was placed on the sample surface with a syringe, and after 10 s, the contact angle was measured in degrees using the software. The data were computed as the average of the right and left contact angles, where a smaller angle indicated greater surface wettability. The test was conducted 6 times for each sample.

### 2.8. Flexural Strength

The specimens were positioned in a universal testing machine (EMIC-DL2000; São José dos Campos, SP, Brazil) for the three-point bending test, where the distance between the supports was 18 mm, and a speed of 5 mm/min was applied at the center of the specimen with a rounded steel rod at the end, with a diameter of 1.2 mm, with a load of 100 kgf until fracture occurred, with values recorded in N, which were converted to MPa to obtain the flexural strength.

$$S=\frac{3FD}{2LH^{2}}$$

where *S* is the flexural strength, *F* is the maximum load (100 kgf), *D* is the distance between supports (18 mm), *L* is the width of the specimen (10 mm), and *H* is the thickness of the specimen (3 mm).

#### 2.8.1. Weibull Analysis

After the three-point bending data were computed, a Weibull analysis was performed using a two-parameter distribution.

$$\begin{array}{c} P_{f}=1-\exp\left|-{\left(\frac{\sigma }{\sigma_{\theta }}\right)}^{m}\right|\\ In\;\left(1-P_{f}\right)=-{\left(\frac{\sigma }{\sigma_{\theta }}\right)}^{m}\\ In\;\left|In\;\left(\frac{1}{1-P_{f}}\right)\right|=nIn\sigma -mIn\sigma_{\theta } \end{array}$$

In this distribution, the characteristic strength σ^θ^ is the location parameter, where a large σ shifts the data to the right, while a small σ shifts the distribution to the left. This parameter corresponds to the strength at which the probability of failure (*P_f_*) corresponds to 63.2%. The Weibull modulus (m) corresponds to the shape of the distribution graph. The higher the distribution, the lower the data dispersion, and the higher the modulus, making the material more reliable.

Significant statistical differences between the characteristic strengths were determined by the overlap of 95% confidence intervals.

#### 2.8.2. Fractographic Analysis

After the three-point flexural strength tests, two fractured samples from each group were analyzed in cross-sections under a scanning electron microscope (Sem, Tescan Vega 3 model, FR, São José dos Campos, Brazil). A voltage of 10 kV was used to obtain images.

### 2.9. Evaluation of Candida Albicans Antibiofilm Activity

The fungal adherence assay used the reference strain *C. albicans* SC 5314 (American Type Culture Collection, Manassas, VA, USA), following the methodology proposed by Rossi et al. [4]. The specimens were divided into 4 groups (*n* = 12 per group): Glaze (G), Alumima + glaze (AlG), Zinc + glaze (ZnG) and Titanium +glaze (TiG). Before the biofilm formation assay, the specimens were sterilized by exposure to ultraviolet radiation for 10 min on each side.

For the assays, *Candida albicans* SC 5314 was plated on Sabouraud dextrose agar and incubated for 24 h at 37 °C under aerobic conditions. After this period, a standardized suspension containing 10^6^ cells/mL in sterile physiological solution (NaCl 0.9%, pH 7.0), was obtained using a spectrophotometer (λ: 550; DO: 0.380). The samples were transferred to 24-well polystyrene plates, where 2000 μL of RPMI broth (added with 2% glucose) and 200 μL of the standardized suspension containing the inoculum were added to each well. The plate was then incubated at 37 °C under shaking (75 rpm) for 24 h. After this step, the culture medium was removed, and the specimens were removed and transferred to 15 mL tubes containing 1000 μL of sterile physiological solution. Each specimen was then sonicated, using two 15 s pulses, with a 5 s interval between them and, after, they were then shaken for 30 s in a vortex shaker, in order to ensure that all cells adhered to the samples were dispersed in the medium. The specimens removed from the tube and the resulting suspension was serially diluted and plated in Sabouraud dextrose agar. The plates were incubated at 37 °C for 24 h, the number of colonies were counted and the number of colony-forming units per milliliter (CFU/mL) were determined.

Two specimens from each group under the described conditions were prepared for scanning electron microscopy analysis. The biofilms were fixed in 2.5% glutaraldehyde for 24 h. After rinsing in physiological solution, dehydration was performed in different concentrations of alcohol (30%, 50%, 70%, 80%, and 90% for 10 min, and 100% for 20 min). Then, the biofilms were dried in an oven at 37 °C for 24 h. The biofilms were metallized (EMITECH SC7620, Sputter Coater, Quorum Technologies, Kent, UK) and observed under a Scanning Electron Microscope (SEM, Vega3, Tescan, Brno, República Tcheca), with images captured and recorded [18].

### 2.10. Statistical Analysis

The integral data were compiled, the means and standard deviations were determined for each analysis conducted. The statistical analysis was made with the SigmaPlot program, by one-way ANOVA (nanoparticle) and Tukey test with a significance level of 0.05.

## 3. Results

### 3.1. X-Ray Diffraction (XRD)

In the X-ray diffractograms, the detected peaks correspond to the crystalline phases characteristic of the nanoparticles deposited on the specimen surface. The TiO_2_ sample exhibited diffraction peaks associated with both anatase and rutile phases, which are the most commonly reported crystalline structures of titanium dioxide and are known to influence photocatalytic and antimicrobial activity. The Al_2_O_3_ sample presented only peaks of the alpha-alumina phase, confirming its high thermal stability and crystalline purity. In turn, the ZnO sample displayed peaks characteristic of the wurtzite structure, which is the thermodynamically stable phase of zinc oxide at room temperature.

**Figure 4:**
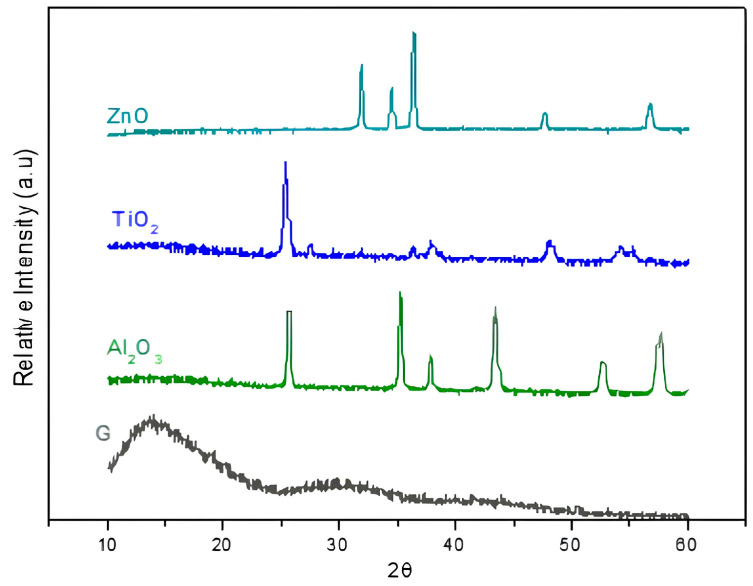
Represents the composition of each group through XRD analysis. G—glaze, Al_2_O_3_—aluminum oxide, TiO_2_—titanium dioxide and ZnO—zinc oxide.

The identification of these crystalline phases is relevant, as the structural arrangement of nanoparticles can directly affect their surface reactivity, stability, and antimicrobial potential. For instance, anatase TiO_2_ has been widely reported to present superior photocatalytic activity compared to rutile, while the wurtzite phase of ZnO has been associated with enhanced interactions with microbial cell membranes. Therefore, the diffractometric findings confirm not only the successful deposition of the nanoparticles but also provide insights into how their intrinsic crystalline structures may contribute to the biological effects observed (Figure 4).

### 3.2. Scanning Electron Microscopy (SEM)

**Figure 5:**
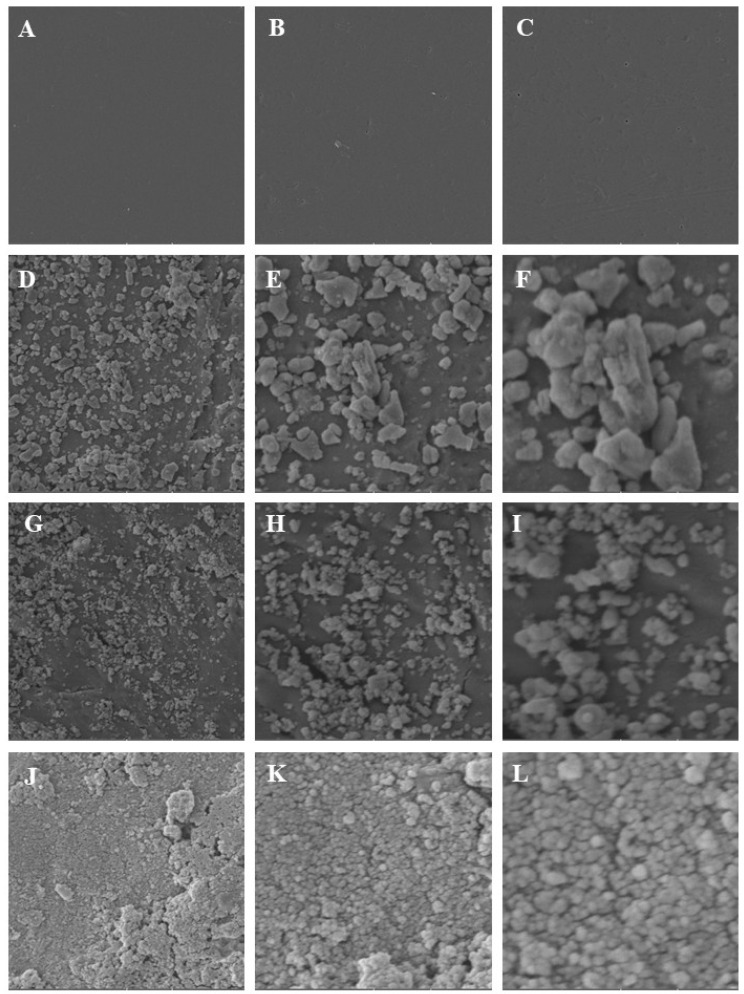
The images are from Scanning Electron Microscopy (SEM). G—glaze (**A**–**C**), AlG—aluminum oxide (**D**–**F**), ZnG—zinc oxide (**G**–**I**) and TiG—titanium dioxide (**J**–**L**). The figures magnification of: (**A**,**D**,**G**,**J**)—2 k, (**B**,**E**,**H**,**K**)—5 k and (**C**,**F**,**I**,**L**)—10 k.

The microscopic analysis revealed different surface aspects that varied according to the surface treatment. The group with only glaze presence (G) on the surface appeared smoother with a more homogeneous aspect. The aluminum oxide group (AlG) exhibited a rougher aspect with greater depth between nanoparticles (100 nm). The zinc oxide (ZnG) and titanium dioxide (TiG) groups showed a surface with nanoparticles (50 nm), well-distributed and less voluminous (Figure 5).

### 3.3. Flexural Strength

The results of the three-point flexural strength test did not reveal any statistically significant differences among the evaluated groups, including the control group (without glaze), the glazed group, and those with the application of alumina, zinc oxide, and titanium dioxide nanoparticles on the surface of heat-polymerized acrylic resin. This finding suggests that the incorporation of these nanoparticles into the glaze layer did not compromise the bulk mechanical integrity of the material.

**Figure 6:**
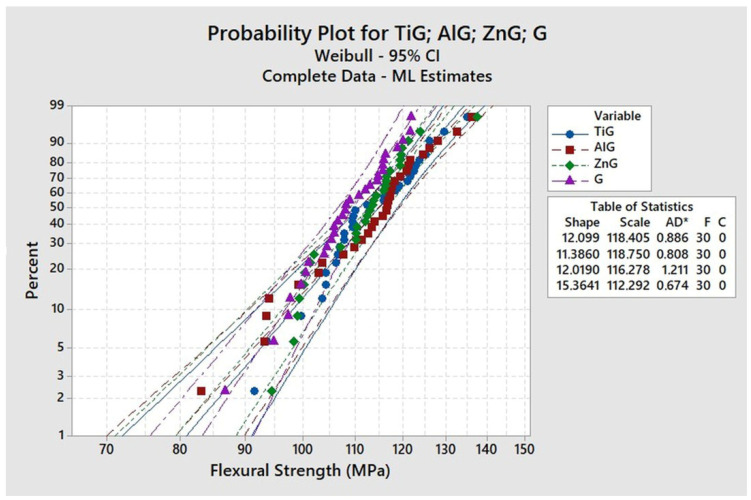
Represents the Weibull analysis, showing similarity in results between groups G, AlG, ZnG e TiG. AD*—According to the Anderson–Darling goodness-of-fit test, the Weibull model showed an acceptable fit to the experimental data for all groups.

The absence of significant alterations in flexural strength is particularly relevant for dental applications, as the mechanical performance of acrylic resins is critical for their long-term clinical use. Maintaining comparable flexural strength across all groups indicates that the surface modification with nanoparticles—although capable of altering surface topography and potentially influencing antimicrobial activity—does not weaken the structural stability of the acrylic resin base (Table 2 and Figure 6).

#### Fractographic Analysis

Fractographic analysis revealed that all groups exhibited internal failures within the material, most likely associated with the presence of bubbles, voids, or other intrinsic contaminations incorporated during the polymerization process of the acrylic resin. These defects are common in heat-polymerized resins and may act as stress concentrators, facilitating crack initiation and propagation under flexural loading.

Importantly, the comparison among groups indicated that the application of glaze, with or without the incorporation of alumina, zinc oxide, or titanium dioxide nanoparticles, did not result in noticeable changes to the fracture pattern. The fractures consistently originated from internal defects rather than from the treated surfaces, suggesting that the surface modifications were not mechanically detrimental to the resin.

**Figure 7:**
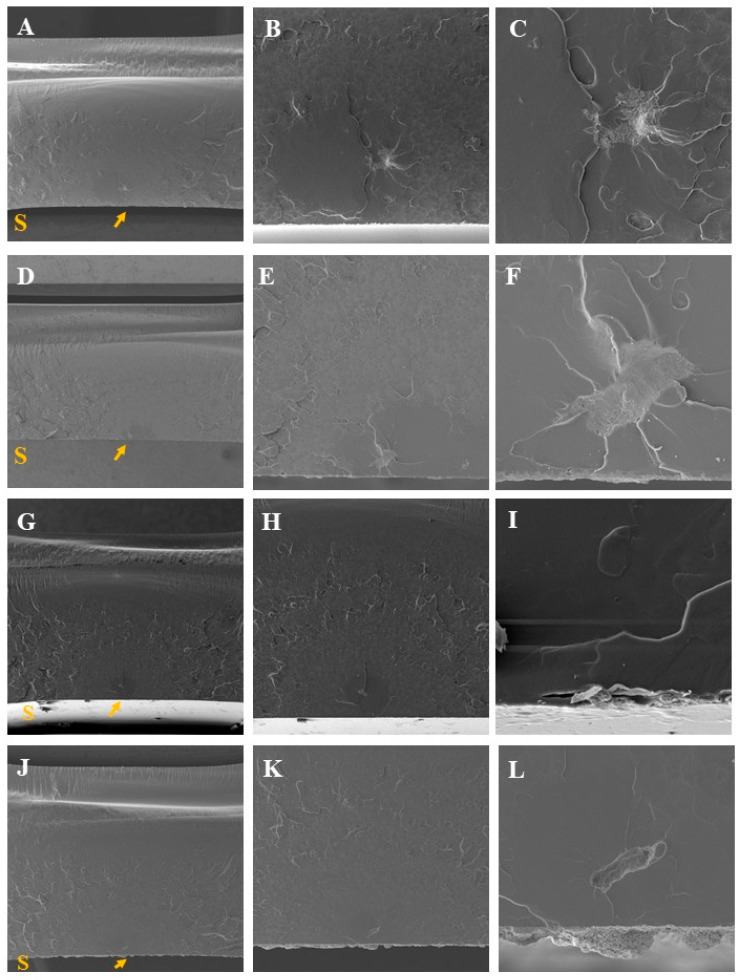
Fractography of the flexural strength test. The yellow arrow indicates the origin of the fracture and S represents the treated surface. The groups are represented by the letters: G—glaze (**A**–**C**), AlG—aluminum oxide (**D**–**F**), ZnG—zinc oxide (**G**–**I**) and TiG—titanium dioxide (**J**–**L**).

These findings support the mechanical results of the three-point flexural strength test, reinforcing that the surface treatments evaluated did not compromise the bulk mechanical integrity of the acrylic resin. From a clinical standpoint, this outcome is significant, as it demonstrates that nanoparticle addition to the glaze layer can be considered without adversely affecting fracture resistance, provided the nanoparticle concentration and application method remain within the evaluated parameters (Figure 7).

### 3.4. Roughness Analysis

The roughness analysis based on the Ra parameter reflects the mean absolute deviation of the surface profile, representing the average height of irregularities along the measured line. In contrast, the Rz parameter evaluates roughness by considering the mean depth between the highest peaks and the lowest valleys within the profile, thus providing a complementary perspective of the surface topography.

In the present study, a statistically significant difference was observed between the control group (G) and the experimental groups treated with nanoparticle-containing glaze. This finding indicates that the incorporation of nanoparticles onto the surface led to an increase in surface roughness. Such an effect is consistent with previous reports, which have demonstrated that nanoparticle deposition can create micro- and nanoscale surface irregularities, altering the topographical features of polymer-based materials (Table 3).

### 3.5. Goniometry

**Figure 8:**
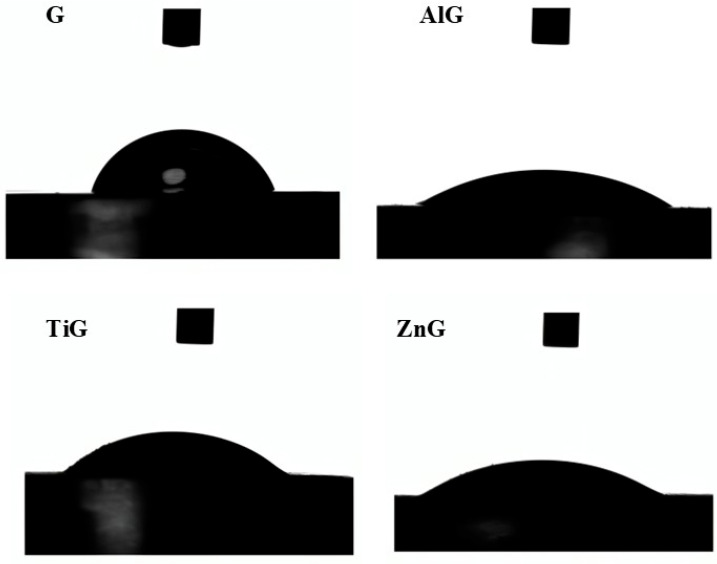
Goniometry test with a water droplet on the treated surface of the polymeric material.

The goniometry test was employed to evaluate the wetting behavior of a water droplet on the different treated and untreated surfaces. The results demonstrated an increase in wettability for the groups treated with zinc oxide (ZnG), titanium dioxide (TiG), and aluminum oxide (AlG), as evidenced by a reduction in the contact angle formed between the droplet and the material surface. This decrease in contact angle indicates that the incorporation of nanoparticles into the glaze layer modified the surface energy, rendering the acrylic resin more hydrophilic (Figure 8).

The test was conducted by calculating the average between the measured angles on the left and right sides of the droplet. A smaller contact angle indicates greater wetting of the surface. Based on the statistical analyses performed using one-way ANOVA and Tukey’s test, it was observed that there was a statistically significant difference between the groups, the Glaze group exhibited less wetting compared to the other groups (Table 4).

### 3.6. Color Measurement

The color measurement analysis was performed directly on the untreated surface, that is, on the opposite side of the treated region, which simulates the clinical perspective of a complete denture—from the polished external surface to the roughened internal surface. The results revealed a statistically significant difference when comparing the group treated with aluminum oxide nanoparticles (AlG) to those treated with zinc oxide (ZnG) and titanium dioxide (TiG).

This finding suggests that the incorporation of different types of nanoparticles into the glaze layer can influence light transmission and reflection through the acrylic resin, ultimately affecting its optical properties. The distinct optical behavior observed among the groups may be explained by the intrinsic refractive indices and particle sizes of the nanoparticles, which determine the degree of light scattering within the material.

From a clinical perspective, such changes are not relevant, since the deposition of nanoparticles would occur in the palatal region of the complete denture, which is not exposed to esthetic demands and therefore does not compromise the overall appearance of the prosthesis (Table 5).

### 3.7. Translucency Measurement

Regarding the translucency parameter, a statistically significant difference was observed when comparing the control group (G) with the experimental groups. The control group exhibited the highest translucency values, whereas the titanium dioxide group (TiG) demonstrated the lowest translucency among all groups.

This reduction in translucency in the presence of nanoparticles may be explained by increased light scattering and absorption caused by the particles embedded in the glaze layer. Titanium dioxide, in particular, is well known for its strong opacifying properties, which likely account for the more pronounced decrease observed in this group.

Although translucency is an important esthetic property for dental materials, it should be noted that in the present study, the nanoparticles were applied to the palatal surface of the complete denture. Consequently, these changes are not expected to have clinical significance, as they would not affect the visible esthetic regions of the prosthesis (Table 6).

### 3.8. Evaluation of Antifungal Activity Candida Albicans Antibiofilm Activity

**Figure 9:**
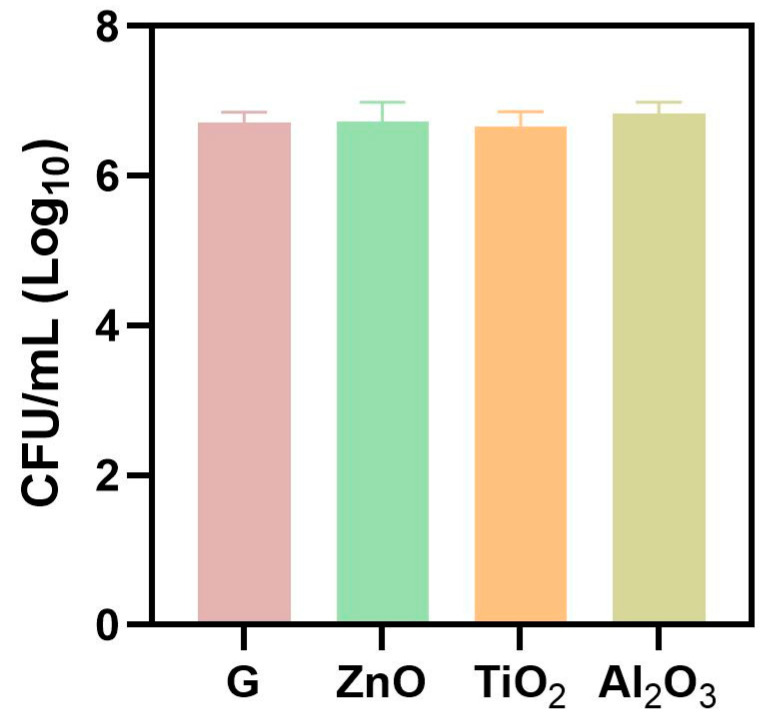
Results for the antifungal effect evaluation, expressed in values of colony forming units per milliliter (CFU/mL), of the following groups: G (acrylic resin + commercial glaze); AlG (acrylic resin+ commercial glaze with alumina oxide); ZnG (acrylic resin+ commercial glaze with zinc oxide and TiG (acrylic resin+ commercial glaze with titanium dioxide).

The number of viable *C. albicans* cells recovered from the samples containing nanoparticles showed no statistically significant difference when compared with the control group. This outcome indicates that fungal proliferation on the sample surfaces was similar across groups, despite the observed increase in surface roughness associated with nanoparticle deposition. These findings suggest that, under the conditions tested, the presence of alumina, zinc oxide, or titanium dioxide nanoparticles in the glaze layer was not sufficient to inhibit fungal growth (Table 7 and Figure 9).

The images obtained through Scanning Electron Microscopy (SEM) of the samples colonized by *C. albicans* revealed denser biofilm formation in the control group, characterized by abundant hyphal growth and surface coverage. In contrast, the groups treated with nanoparticle-containing glaze exhibited visibly reduced hyphal aggregation and lower biofilm density on the surface.

**Figure 10:**
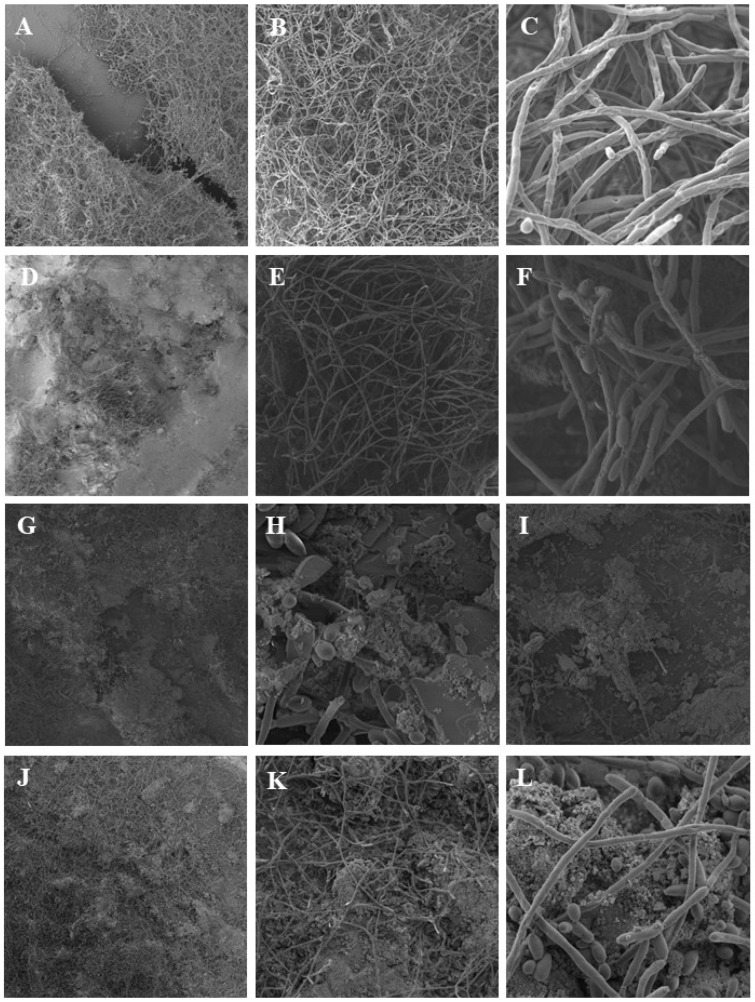
Scanning Electron Microscopy (SEM) of the sample with the fungus *C. albicans,* with a reduction in hyphal form in the treated groups. G—glaze (**A**–**C**), AlG—aluminum oxide (**D**–**F**), ZnG—zinc oxide (**G**–**I**) and TiG—titanium dioxide (**J**–**L**). The figures have magnifications of: (**A**,**D**,**G**,**J**)—0.2 k, (**B**,**E**,**H**,**K**)—1 k, (**C**,**F**,**I**,**L**)—5 k.

These morphological observations suggest that, although the quantitative analysis of viable cell counts did not demonstrate statistically significant differences, the presence of nanoparticles may have influenced the pattern of fungal colonization. The reduction in hyphal structures in the treated groups indicates a possible interference with the fungal transition from yeast to hyphal form, which is a key virulence factor for *C. albicans* biofilm development (Figure 10).

## 4. Discussion

The application of treatments on the surface of restorative materials is being increasingly researched with the purpose of improving or enhancing properties already present in the materials. Such is the case of the present study, which sought to improve the properties of thermally activated acrylic resin commonly used in the fabrication of complete dentures. The pursuit of an increase in the surface quality of the thermally activated acrylic resin (TAAR) was investigated in this study through the application of nanoparticles such as zinc oxide (ZnO) and titanium dioxide (TiO_2_) in the glaze applied over the acrylic resin. This was aimed at promoting: antimicrobial action against *C. albicans*, enhanced surface wetting to facilitate better peripheral sealing of the prostheses and preservation of material strength.

One of the results obtained in the present study was an increase in surface roughness (parameters of height and depth) for the groups with surface treatment when compared to the control group (G). This increase in roughness, together with the nanometric characteristics of the nanoparticles, can lead to a proportional increase in the surface area, consequently enhancing surface wetting capacity. Clinically, increased contact of saliva with the surface of the prosthesis will allow for greater stability of the prosthetic piece, due to the sealing that occurs through the presence of saliva between the inner surface of the prosthesis and the mucosa [15].

Beger et al. [19] reported that the roughness of the inner portion of a prosthesis, without polishing, is approximately 4.588 µm. This data shows that the roughness value found for the groups with nanoparticles on the surface, in the present study, was lower than the roughness value of the inner portion of a prosthesis without polishing. Thus, it can be observed that the application of these nanoparticles to the inner portion of the prosthesis would not necessarily increase the roughness of the prosthesis and consequently its microbial aggregation capacity. However, due to the structural characteristics of the nanoparticles, an increase in the surface wetting capacity of the complete dentures would be possible.

The surface roughness found for the treated groups was higher than when compared to the control group with glaze, which promotes an increase in surface energy and better surface wetting that can lead to greater stability of the prosthesis. This is a beneficial factor in certain clinical cases with lower retention of the prosthetic piece due to a smaller height and width of the ridge [3,15].

Another result obtained was the decrease in translucency and color alteration of the groups treated with nanoparticles as compared to the control, which does not interfere with the success of the prosthetic treatment, considering that the clinical application of nanoparticles would be in the palate region or in areas that do not aesthetically compromise the prosthesis. Thus, the decrease in translucency and color alteration would not aesthetically influence the prosthetic outcome, as it would not be applied in an aesthetic region [20,21].

In the present study, the incorporation of alumina, zinc, and titanium nanoparticles into the glaze of acrylic resin did not enhance antifungal activity. This outcome may be attributed either to insufficient nanoparticle concentration relative to the sample surface area or to the increase in surface roughness observed in the treated groups when compared with the control group, which received only the glaze application. Other study has demonstrated that a concentration of 1% TiO_2_ exhibits antimicrobial activity, whereas 0.5% does not show such potential [10], the same response was found for the concentration of 0.8 wt% ZnO and TiO_2_ nanoparticles [22]. That way, the antifungal efficacy against different *Candida* species varies depending on the nanoparticle concentration [10,17]. Furthermore, another study indicated that higher concentrations of ZnO are required to suppress *Candida albicans* proliferation, with significant activity observed at 5 wt%, 10 wt%, and 20 wt% [23]. Therefore, it is plausible that an increased nanoparticle concentration in the present study might lead to measurable fungal inhibition.

Unfortunately, increasing the nanoparticle concentration in the present study proved to be unfeasible, as a higher amount would be difficult to adhere to the surface area, given the limited quantity of glaze applied. Nevertheless, although no statistically significant differences were observed among the groups, scanning electron microscopy images revealed a marked reduction in bacterial proliferation in the groups containing ZnO and TiO_2_ (Figure 10).

Additionally, it is possible that there was an interaction between the glaze and the nanoparticles that were incorporated on its surface, thereby hindering fungal inhibition. This characteristic would not be entirely surprising, considering that a previous study Rossi et al. [4] were conducted with the application of nanoparticles inside the glaze liquid, where there was no antimicrobial action due to the coating of nanoparticles by the liquid, preventing direct contact with the fungus. Further, it is well established in the literature that the heterogeneous surface and area are important factors in determining the antimicrobial properties of a nanoparticle, such as zinc oxide [16]. This oxide can exhibit different morphologies and inhibitions of bacteria growth. Even though similar phenomenon can occur with fungal species, studies on these microorganisms, such as *C. albicans*, are still scarce.

Regarding the Scanning Electron Microscopy (SEM) images of the samples subjected to the antimicrobial test, it is evident that, despite the lack of statistically significant differences between the groups, the control group exhibited a greater densification of *C. albicans* in the hyphal state [24]. In contrast, the treated groups exhibited greater spreading and less agglomeration of the microorganism on the surface, this observation may reflect morphological changes or cellular dispersion rather than an actual reduction in microbial density Additionally, an analysis was conducted using SEM to assess the area and height of nanoparticles present on the surface to quantify the nanoparticle concentration on the surface. This allowed us to understand that the incomplete coverage of the sample surface by nanoparticles at the micrometer scale, and consequently the low nanoparticles’ concentration may have influenced antimicrobial action [25,26].

Pasquet et al. [27] suggested that the antimicrobial action of zinc nanoparticles against fungi is lower compared to bacteria. Additionally, the fungistatic effect of zinc oxide to maintain the initial population rather than reducing it was also observed previously [14]. Conversely, Eskandari et al. [28] demonstrated fungicidal action of this nanoparticle against *C. albicans*, leading to a decrease in fungal growth. Thus, it is evident that more consolidated studies are still needed to prove the antifungal action of ZnO against *C. albicans*, as smaller nanoparticles potentially exhibit better antimicrobial action [17].

Regarding titanium dioxide nanoparticles, studies have shown a 35% inhibition in the adherence of *C. albicans* in its hyphal form [28]. Titanium dioxide nanoparticles exhibit better antimicrobial action when in the form of anatase and rutile. The present study is the first, within the literature found, to apply this commercial nanoparticle in a dental polymeric material. Aiming for the clinical applicability of this surface treatment, it is necessary to analyze the individuality of each clinical case to better indicate its application [9,29].

Based on the discussion presented in the article, it can be concluded that the application of zinc oxide (ZnO) and titanium dioxide (TiO_2_) nanoparticles on the surface of thermally activated acrylic resin shows potential for enhancing properties relevant to the fabrication of complete dentures. The study focused on improving surface quality through the use of nanoparticles, with aims such as promoting antimicrobial action, enhancing wettability, and preserving material strength.

The results of the study indicated that while the surface roughness increased for the treated groups compared to the control group, this increase could lead to enhanced surface wetting capacity, potentially improving stability of the prosthetic piece. The decrease in translucency and color alteration observed with nanoparticle treatment did not seem to impact the overall success of the prosthetic treatment, particularly in non-aesthetically sensitive areas of the prosthesis.

## 5. Conclusions

Regarding antifungal activity, no statistically significant differences were observed between the control and treated groups. This outcome may be attributed to factors such as insufficient nanoparticle concentration or interactions between the glaze and the nanoparticles that may have limited fungal inhibition. Nevertheless, microscopy images revealed a reduction in the presence of hyphae on surfaces treated with zinc oxide and titanium dioxide. Future studies employing higher nanoparticle concentrations may yield different results.

Overall, this study provides valuable insights into the potential benefits and limitations of incorporating nanoparticles into dental materials. Further research focusing on optimizing nanoparticle concentrations, enhancing surface coverage, and elucidating the interactions between nanoparticles and substrate materials may contribute to improving the clinical effectiveness of these treatments.

## Figures and Tables

**Table 1:** Antimicrobial properties and mechanisms of action of ZnO and TiO_2_ nanoparticles, through different modes of analysis.

Authors	Nanoparticles	How Was It Analyzed?	Mechanism of Action
Kairyte et al., 2013 [14]	ZnO	Antimicrobial activity—reduction in Colony forming unit (CFU)	Production of ROS causes damage to the cell membrane, and with photoactivation, bubbles are formed on the surface of the microorganism, which leads to cell shrinkage and the release of intracellular components. Electrostatic interaction of NPs with the cell wall; production of hydrogen peroxide.
Foster et al., 2011 [9]	TiO_2_	Antimicrobial activity—reduction in Colony forming unit (CFU)	Degradation of the cell wall and cytoplasmic membrane occurs because of the production of ROS such as hydrogen peroxide and hydroxyl radicals. Disruption of the phospholipid bilayer can also occur.
Darwish et al., 2019 [15]	TiO_2_	Antimicrobial activity—reduction in Colony forming unit (CFU)	Reactive oxygen species (ROS) cause irreversible damage to the cell surface.
Hosseini et al., 2018 [16]	ZnO	Microbial adhesion test (2 h) Biofilm formation analysis (48 h) Determination of Minimum Inhibitory Concentration (MIC)	Destroys the integrity of the cell membrane through the production of reactive oxygen species (ROS)
da Silva et al., 2019 [17]	ZnO	Disk diffusion Determination of Minimum Inhibitory Concentration (MIC)	Production of ROS. Loss of cellular integrity, due to contact of the ZnO NP with the cell wall. Release of Zn^2+^ ions. Photoconductivity that occurs through a photoinduction process, in which, when subjected to UV light, the antimicrobial action is optimized owing to the improvement in conductivity.

**Table 2:** Mean and standard deviation for strength analysis, Weibull modulus (m) and characteristic strength (σθ).

Groups	N	Mean (MPa) ± Standard Deviation	m CI (95%)	σθ CI (95%)
G	30	108.54 ± 8.36 A	15.3 A (11.6–20.3)	112.3 A (109.5–115.1)
AlG	30	113.60 ± 11.95 A	11.4 A (8.6–15.0)	118.7 A (115.0–122.74)
ZnG	30	111.98 ± 9.27 A	12.0 A (9.3–15.4)	116.3 A (112.6–120.0)
TiG	30	113.66 ± 10.41 A	12.1 A (9.2–16.0)	118.4 A (114.7–122.1)

Different letters indicate statistically significant differences among the study groups (One-way ANOVA and Tukey’s test 5%, *p* = 0.221).

**Table 3:** Measurement of roughness (Ra and Rz) was performed among the groups.

Groups	N	Ra (µm) ± Standard Deviation	Rz (µm) ± Standard Deviation
G	10	0.2695 ± 0.07 A	1.044 ± 0.49 A
AlG	10	3.792 ± 1.36 B	24.09 ± 6.64 C
ZnG	10	3.230 ± 0.85 B	19.90 ± 3.54 B
TiG	10	3.813 ± 0.94 B	22.86 ± 3.23 BC

Different letters indicate statistically significant differences among the study groups (One-way ANOVA and Tukey’s test 5%, *p* < 0.0001).

**Table 4:** Represents the results of goniometry for the groups.

Groups	N	Mean ± Standard Deviation
G	6	74.9 ± 2.68 A
AlG	6	40.25 ± 6.05 B
ZnG	6	39.75 ± 1.61 B
TiG	6	41.75 ± 0.41 B

Different letters indicate statistically significant differences among the study groups (One-way ANOVA and Tukey’s test 5%, *p* = 0.001).

**Table 5:** Color measurement analysis.

Groups	N	Mean ± Standard Deviation
AlG	10	7.348 ± 3.573 A
ZnG	10	8.028 ± 4.112 B
TiG	10	9.189 ± 3.387 B

Different letters indicate statistically significant differences among the study groups (One-way ANOVA and Tukey’s test 5%, *p* = 0.539).

**Table 6:** Translucency measurement analysis.

Groups	N	Mean ± Standard Deviation
G	10	38.929 ± 4.368 A
AlG	10	20.555 ± 5.780 B
ZnG	10	21.910 ± 5.273 B
TiG	10	14.720 ± 2.817 C

Different letters indicate statistically significant differences among the study groups (One-way ANOVA and Tukey’s test 5%, *p* ≤ 0.001).

**Table 7:** Analysis of *C. albicans* antibiofilm effect of the groups: G (acrylic resin + commercial glaze); AlG (acrylic resin+ commercial glaze with alumina oxide); ZnG (acrylic resin+ commercial glaze with zinc oxide) and TiG (acrylic resin+ commercial glaze with titanium oxide).

Groups	N	Mean log^10^ ± Standard Deviation
G	12	6.711 ± 0.11
AlG	12	6.827 ± 0.08
ZnG	12	6.723 ± 0.22
TiG	12	6.663 ± 0.18

One-way ANOVA and Tukey’s test with 5% significance level (*p* = 0.218).

## Data Availability

The raw data supporting the conclusions of this article will be made available by the authors on request.
